# Draft Genome Sequence of a Multiresistant Bovine Isolate of *Staphylococcus lentus* from Tanzania

**DOI:** 10.1128/genomeA.01345-16

**Published:** 2016-12-01

**Authors:** Jeremiah Seni, Stephen E. Mshana, Felician Msigwa, Mecky Matee, Humphrey Mazigo, Julian Parkhill, Mark A. Holmes, Gavin K. Paterson

**Affiliations:** a Department of Microbiology, Immunology, Weill Bugando School of Medicine, Tanzania; b Department of Microbiology, Immunology, School of Medicine, Muhimbili University of Health and Allied Sciences, Tanzania; c Department of Parasitology and Entomology, Weill Bugando School of Medicine, Tanzania; d The Wellcome Trust Sanger Institute, Wellcome Trust, Genome Campus, Hinxton, United Kingdom; e Department of Veterinary Medicine, University of Cambridge, Cambridge, United Kingdom; f Royal (Dick) School of Veterinary Studies and Roslin Institute, The University of Edinburgh, Easter Bush Campus, United Kingdom

## Abstract

We report here the draft genome sequence of a *Staphylococcus lentus* isolate, 050AP, collected in Tanzania from a swab of healthy bovine perineum. The draft genome sequence contained 2.72 Mbp and 2,750 coding sequences with a G+C content of 31.7%.

## GENOME ANNOUNCEMENT

*Staphylococcus lentus* is a member of the *Staphylococcus sciuri* group which also comprises *S. sciuri*, *Staphylococcus vitulinus*, *Staphylococcus fleurettii*, and *Staphylococcus stepanovicii* (1, 2). This group is part of the normal skin and mucosal flora in a wide range of animals, and while not frequently associated with disease, members of the *S. sciuri* group have been isolated from various human and veterinary infections (1, 2). They have furthermore been implicated as a reservoir for virulence and resistance gene exchange with other staphylococci (2). In this study an isolate of *S. lentus*, 050AP, collected from a perineum swab of a healthy Friesian-Jersey mixed breed dairy cow in Nyakato, Tanzania in April 2014 was genome sequenced using an Illumina HiSeq 2000. To our knowledge this is the first veterinary *S. lentus* genome to be reported.

Genome assembly was performed using Velvet software (3), and resulted in an assembly consisting of 37 contigs with a *N*_50_ of 119,892 bp which was automatically annotated using Prokka (4). The resultant 050AP draft genome was 2,719,515 bp with a G+C content of 31.7% and contained 2,750 predicted protein-coding sequences. The macrolide resistance gene *mph*(C) and tetracycline resistance gene *tet*(K) were identified by ResFinder version 2.1 (5). In the case of *mph*(C) (locus tag: SAMEA3109314_01885) the best full length BLAST match in the nucleotide collection is a 91.9% identify match to *mph*(C) in *Staphylococcus aureus* (CP017097.1). Thus, 050AP appears to encode a novel *mph*(C) variant which may be the same or related to a variant reported as partial coding sequences in *S. lentus* from free-living small mammals in Poland (6). A single nucleotide deletion causes a frameshift mutation in *tet*(K) leading to a predicted protein of at least 419 amino acids versus the typical 296 amino acids. However, with the gene located at the end of a contig the exact size of *tet*(K) in 050AP is uncertain from these sequence data. Phenotypically 050AP was resistant to erythromycin, clindamycin, tetracycline, ciprofloxacin, fusidic acid, oxacillin, and trimethoprim as assessed by Vitek-2 using card AST-P620 (bioMérieux, Basingstoke, United Kingdom) but susceptible to cefoxitin (screen), chloramphenicol, daptomycin, gentamicin, linezolid, mupirocin, penicillin, teicoplanin, tigecycline, and vancomycin. The availability of this genome for comparative analysis with other staphylococcal genomes will provide insights into the biology of the *S. sciuri* group and their role as commensals and pathogens.

### Accession number(s).

This whole-genome shotgun project has been deposited in DDBJ/EMBL/GenBank under the accession number FMRW01000000. The version described in this paper is the first version, FMRW01000000.1.
